# The relationship between digital health literacy and health anxiety among Chinese older adults: the role of aging attitudes and physical activity

**DOI:** 10.3389/fpubh.2026.1751272

**Published:** 2026-02-13

**Authors:** Changzhou Chen, Xingyi Li, Sen Li

**Affiliations:** 1 School of Physical Education, Shanghai University of Sport, Shanghai, China; 2 Department of Sport and Health, Shinhan University, Uijeongbu-si, Gyeonggi-do, Republic of Korea; 3 School of Physical Education and Health, Shanghai Lixin University of Accounting and Finance, Shanghai, China

**Keywords:** aging attitudes, digital health literacy, health anxiety, older adults, physical activity

## Abstract

**Objective:**

Against the backdrop of rapid population aging and increasing digitalization, health anxiety has emerged as a significant public health concern affecting the physical and psychological well-being of older adults in China. Digital health literacy, defined as an individual’s ability to access, understand, and evaluate health-related information in digital environments, may play a crucial role in shaping health anxiety. However, the underlying mechanisms linking digital health literacy to health anxiety remain insufficiently examined. Therefore, this study aimed to investigate the association between digital health literacy and health anxiety among Chinese older adults, with particular attention to the mediating roles of aging attitudes and physical activity.

**Methods:**

A total of 535 Chinese older adults were recruited using a convenience sampling strategy. Validated standardized questionnaires were used to assess digital health literacy, aging attitudes, physical activity levels, and health anxiety. Correlation and regression analyses were conducted, and the mediating effects of aging attitudes and physical activity, as well as their chain mediation effect, were tested using the Bootstrap method.

**Results:**

Digital health literacy was significantly and negatively associated with health anxiety (*β* = −0.239, *p* < 0.01). Aging attitudes [95% CI (−0.080, −0.025)] and physical activity [95% CI (−0.029, −0.002)] independently mediated the relationship between digital health literacy and health anxiety. Furthermore, a significant chain mediation effect of aging attitudes and physical activity was observed [95% CI (−0.008, −0.001)].

**Conclusion:**

Digital health literacy not only directly alleviates health anxiety among older adults but also indirectly reduces anxiety by fostering more positive aging attitudes and promoting higher levels of physical activity. These findings elucidate key psychological and behavioral mechanisms underlying health anxiety in digital contexts and provide important implications for public health interventions. Integrated strategies combining digital skills training, positive aging education, and physical activity promotion may be effective in enhancing the mental and physical health of older adults.

## Introduction

Against the backdrop of accelerating global population aging, promoting the physical and psychological well-being of older adults has become a major public health concern in the twenty-first century (1). As the country with the largest aging population worldwide, China is experiencing a particularly rapid and extensive demographic shift. According to data from the Seventh National Population Census, the number of individuals aged 60 years and above has reached 264 million, accounting for 18.70% of the total population (2). Alongside aging-related physiological decline, the high prevalence of chronic diseases, and transitions in social roles, mental health challenges among older adults have become increasingly prominent (3). Among these challenges, health anxiety has emerged as a critical psychological risk factor affecting quality of life and contributing to rising healthcare utilization. Health anxiety refers to an individual’s tendency to interpret ordinary or ambiguous bodily sensations in a catastrophic manner and to become excessively preoccupied with concerns about personal health (4). Such anxiety not only increases psychological distress but may also lead to excessive medical consultations and unnecessary healthcare use, thereby placing additional strain on public healthcare systems (5). Consequently, a systematic and in-depth examination of health anxiety among older adults is of substantial practical and societal significance.

Existing research has largely drawn on cognitive–behavioral frameworks to explain the development of health anxiety, emphasizing individuals’ propensity to misinterpret ambiguous bodily sensations as indicators of serious illness, which subsequently triggers anxiety responses (6). In studies focusing on older adults, two primary lines of inquiry have predominated. First, at the cognitive–psychological level, negative aging attitudes have been consistently shown to be positively associated with health anxiety (7). Second, at the social–environmental level, factors such as insufficient social support and heightened loneliness, which are prevalent among older populations, have also been identified as important predictors of health anxiety (8, 9). However, much of this literature remains rooted in traditional psychosocial perspectives and pays limited attention to emerging cognitive and behavioral mechanisms operating within digital contexts. With the widespread adoption of the internet and smart devices among older adults, modes of accessing health information have undergone fundamental changes. Differences in digital health literacy result in markedly divergent interpretations of and responses to online health information: while some older adults are able to actively screen, evaluate, and apply health-related information, others may become overwhelmed, leading to excessive worry and anxiety. This contrast suggests that conventional psychosocial explanatory frameworks may be insufficient to fully capture the mechanisms underlying health anxiety in the digital era. In recent years, digital health literacy—defined as an individual’s ability to access, understand, evaluate, and apply health information within digital environments—has been increasingly recognized as a key personal resource for maintaining health in digitally mediated societies (10). Higher levels of digital health literacy may enable older adults to identify misleading or exaggerated health information, thereby reducing unnecessary anxiety and concern (11), whereas insufficient literacy may increase the likelihood of misinterpreting online information and exacerbate health anxiety (12). Despite its growing relevance, systematic empirical research on digital health literacy among older adults in China remains limited. This gap is particularly salient given the pronounced digital divide in Chinese society, where disparities related to urban–rural residence, educational attainment, and generational differences contribute to widespread deficits in digital skills and information utilization among older adults (13). Such disparities may further amplify the risk of health anxiety in this population. Therefore, examining the relationship between digital health literacy and health anxiety not only addresses an important gap in the existing literature but also responds directly to the practical challenges posed by population aging in contemporary Chinese society.

Furthermore, the association between digital health literacy and health anxiety may not be limited to a single direct pathway but may also operate indirectly through specific cognitive and behavioral processes. First, aging attitudes, defined as individuals’ cognitive evaluations of the aging process, represent a key psychological mechanism influencing mental health outcomes in later life (14). Positive aging attitudes can reduce older adults’ tendency to catastrophize normal age-related bodily changes, thereby alleviating health anxiety (8, 15). In this sense, digital health literacy may not only facilitate the development of more accurate and scientific health beliefs but also buffer health anxiety by fostering more positive aging attitudes. Second, physical activity constitutes a core lifestyle behavior for maintaining physical and psychological health among older adults. Regular physical activity has been shown to enhance physical functioning and sleep quality (16), as well as to reduce stress and anxiety (17). Higher levels of digital health literacy may further enable older adults to access exercise-related knowledge, programs, and resources through online platforms, thereby promoting greater engagement in physical activity (18).

Based on these considerations, the present study aims to systematically examine the relationship between digital health literacy and health anxiety among older adults in China, with a particular focus on the mediating roles of aging attitudes and physical activity. By incorporating emerging mechanisms embedded in digitally mediated social contexts, this study seeks to extend existing explanatory frameworks of health anxiety and to provide empirical evidence and practical implications for enhancing digital health literacy and promoting positive aging in the Chinese older population.

## Theoretical framework

### Digital health literacy and health anxiety in older adults

According to Conservation of Resources theory, individuals tend to protect existing resources, acquire new resources, and prevent resource loss when confronted with threatening events in order to maintain psychological equilibrium (19). Within this theoretical framework, digital health literacy can be conceptualized as a critical cognitive resource that enables older adults to manage health-related information more effectively and enhances their sense of self-efficacy (20). Health anxiety is fundamentally characterized by uncertainty regarding bodily sensations and a tendency toward catastrophic interpretations of physical symptoms (6). Digital health literacy serves as a key tool for managing such uncertainty. Higher levels of digital health literacy enhance older adults’ ability to access, understand, and evaluate health information (10), thereby narrowing information gaps and reducing cognitive load and perceived risk when facing potential illness. As a result, anxiety arising from informational uncertainty may be alleviated (21). Moreover, within traditional healthcare models, older adults are often positioned as passive recipients of medical information and services. In contrast, digital health literacy empowers older adults to actively engage in self-monitoring and health management through digital tools. This active engagement reflects greater autonomy and personal agency (22) and contributes to enhanced self-efficacy. Increased self-efficacy, in turn, represents a crucial psychological foundation for older adults to cope with fears related to aging and health anxiety (20).

Taken together, the level of digital health literacy may shape older adults’ overall resource status when confronting health-related threats. Higher digital health literacy implies the availability of greater cognitive resources, enabling individuals to more proactively interpret health information, assess risks, and adopt appropriate coping strategies, thereby helping to maintain psychological balance. Conversely, lower levels of digital health literacy are associated with more limited cognitive resources, making individuals more vulnerable to uncertainty and worry when encountering complex health information, which may ultimately increase the risk of health anxiety. Although theoretical reasoning and some empirical evidence support these mechanisms, systematic validation within Chinese older populations remains limited. Accordingly, the present study proposes the following hypothesis:

H1: Digital health literacy is negatively associated with health anxiety among older adults.

### The mediating role of aging attitudes

Aging attitudes, defined as individuals’ beliefs, emotions, and evaluations regarding their own aging and the aging process in general, represent a deep-seated cognitive factor influencing psychological adaptation and health-related behaviors in later life (23). Previous research has shown that positive aging attitudes such as viewing later life as a period of continued growth and the accumulation of wisdom are closely associated with more favorable health outcomes and longer life expectancy (24). In contrast, negative aging attitudes that equate aging with illness and functional decline have been consistently identified as important predictors of psychological problems, including depression and anxiety (23, 25). From the perspective of Conservation of Resources theory, aging attitudes can be understood as indicators of the availability or depletion of psychological resources. Hobfoll (19, 26) conceptualized psychological resources as capacities that help individuals maintain a positive self-concept, a sense of meaning, and perceived control. Positive aging attitudes embody these core psychological resources, enabling older adults to perceive aging as a natural and manageable stage of the life course rather than as an inevitable loss or threat (15). Given that catastrophic interpretations of bodily changes constitute a central cognitive basis of anxiety responses (6), positive aging attitudes may reduce older adults’ tendency toward such catastrophic appraisals and, consequently, alleviate health anxiety.

Within this theoretical framework, psychological resources are regarded as fundamental for coping with challenges, as their availability shapes individuals’ subjective appraisals of stressors and subsequent emotional responses. As an important cognitive resource, digital health literacy extends beyond technical skills related to accessing health information and encompasses individuals’ capacities to understand, evaluate, and integrate health-related information (10). Hobfoll et al. (27) further emphasized that individuals mobilize existing cognitive resources to identify, interpret, and respond to potential resource threats, thereby facilitating the acquisition and accumulation of other core psychological resources and enhancing psychological adaptation under stress. Specifically, older adults with higher levels of digital health literacy are more likely to actively seek and utilize health information through digital platforms and to engage in online health-related interactions (28). Such digital engagement may reduce negative aging stereotypes by updating health-related knowledge and beliefs (29), while also strengthening perceived social connectedness (30). As these cognitive and social experiences accumulate, individuals’ understandings of aging may gradually shift from viewing it as an unavoidable decline or loss to perceiving it as a stage of life that can be understood, managed, and adapted to, thereby fostering a more positive aging-related cognitive framework (15, 31).

Taken together, within the Conservation of Resources theory framework, positive aging attitudes not only reflect a state of abundant psychological resources but may also serve as a bridging mechanism between cognitive resources and emotional outcomes. On the one hand, higher digital health literacy may help older adults revise negative aging stereotypes and develop more positive aging-related cognitions. On the other hand, these positive aging attitudes may reduce catastrophic interpretations of bodily changes, thereby buffering health anxiety. Although previous studies have separately examined the associations among digital health literacy, aging attitudes, and health anxiety, the psychological pathways through which digital health literacy influences health anxiety in older adults remain insufficiently tested. Accordingly, the present study proposes the following hypothesis:

H2: Aging attitudes mediate the relationship between digital health literacy and health anxiety among older adults.

### The mediating role of physical activity

Physical activity is widely recognized by the World Health Organization as a key behavior for promoting health across the lifespan, preventing chronic diseases, and improving psychological well-being (32). According to Conservation of Resources theory, one of the core principles guiding human behavior is individuals’ tendency to invest resources continuously in order to prevent resource loss and acquire new resources (27). Within this framework, physical activity can be conceptualized as a prototypical form of indirect resource investment. Through sustained engagement in physical activity, individuals gradually accumulate both physiological and psychological resources, thereby supporting mental health.

On the one hand, higher levels of digital health literacy may enable older adults to access scientific and personalized exercise-related information. For example, older adults can use mobile applications to learn exercise routines tailored to their physical conditions (e.g., fitness exercises or Tai Chi) or employ wearable devices to monitor physical activity data, which may motivate and guide regular participation in physical activity (33, 34). From the perspective of Conservation of Resources theory, this process reflects individuals’ use of cognitive resources to make informed resource investment decisions aimed at preventing potential losses in health-related resources. On the other hand, regular physical activity provides older adults with a sustainable pathway for resource accumulation. Existing evidence indicates that beyond improvements in physical fitness and functional health, regular physical activity is associated with lower risks of loneliness and social isolation, as well as higher levels of social support key psychosocial protective factors in later life (35). Moreover, physical activity has been positively linked to self-efficacy (36), thereby providing important psychological and social resources that facilitate psychological adaptation. The synergistic accumulation of physical, psychological, and social resources generated through physical activity may reduce individuals’ sensitivity to health-related threats and, in turn, enhance overall psychological well-being.

Although a substantial body of evidence has demonstrated the anxiety-reducing effects of physical activity from physiological perspectives (17), Conservation of Resources theory highlights a deeper explanatory mechanism: physical activity may alleviate health anxiety by weakening expectations of health-related resource loss through continuous resource regeneration and accumulation. Taken together, physical activity is likely to constitute a critical behavioral pathway linking digital health literacy to health anxiety. Specifically, digital health literacy may alleviate health anxiety among older adults by increasing both the level and the scientific quality of engagement in physical activity. Accordingly, the present study proposes the following hypothesis:

H3: Physical activity mediates the relationship between digital health literacy and health anxiety among older adults.

### The chain mediating role of aging attitudes and physical activity

Beyond examining the independent mediating roles of aging attitudes and physical activity in the association between digital health literacy and health anxiety, the present study further proposes that these two factors may constitute a sequential psychological–behavioral pathway. From the perspective of Conservation of Resources theory, this pathway illustrates how digital health literacy, as a cognitive resource, may extend from the cognitive domain to psychological and behavioral domains through continuous resource transformation and investment, thereby offering a potential explanation for the resource gain spiral. Specifically, digital health literacy may contribute to the formation of more positive aging attitudes, which in turn promote higher levels of physical activity, ultimately being associated with lower levels of health anxiety. First, positive aging attitudes are not limited to subjective cognitive evaluations of aging (i.e., how individuals understand the aging process) but also encompass perceptions of personal competence and expectations of controllability (15). Such cognitions have been shown to predict more proactive health self-management behaviors and to be closely associated with higher levels of positive affect among older adults (37). In other words, when older adults no longer perceive aging as an irreversible decline but rather as a life stage in which physical functioning and independence can still be maintained through personal effort, they are more inclined to engage in physical activity to preserve their physical and psychological capacities. This reasoning is consistent with research on positive aging and successful aging, which suggests that positive self-perceptions of aging enhance action readiness and self-efficacy, thereby facilitating sustained participation in physical activity (38, 39). Second, increased engagement in physical activity itself is closely linked to lower levels of anxiety. A substantial body of empirical evidence indicates that regular physical activity can reduce anxiety symptoms and health-related worries through multiple pathways, thereby buffering health anxiety (40). Studies focusing specifically on older adults have further demonstrated that maintaining regular physical activity is associated with greater well-being and lower levels of health anxiety (41, 42).

Taken together, these findings suggest that digital health literacy may initiate a sequential process in which enhanced cognitive resources foster more positive aging attitudes, which subsequently encourage greater engagement in physical activity, ultimately contributing to reduced health anxiety. Accordingly, the present study proposes the following hypothesis:

H4: Aging attitudes and physical activity chain mediate the relationship between digital health literacy and health anxiety among older adults.

### The current study

**Figure 1:**
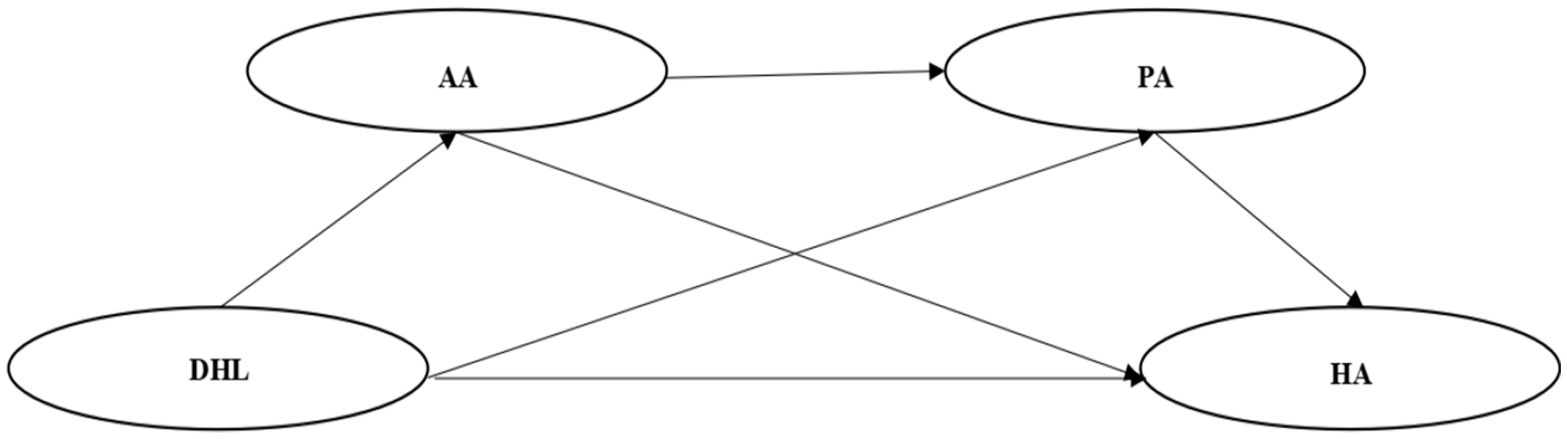
Concept model. DHL, digital health literacy; HA, health anxiety; AA, aging attitudes; PA, physical activity.

Taken together, the present study aims to examine the association between digital health literacy and health anxiety among older adults in China, as well as the underlying psychological and behavioral mechanisms. As illustrated in Figure 1, an overall conceptual model was developed, and the following hypotheses were proposed:

H1: Digital health literacy is negatively associated with health anxiety among older adults.

H2: Aging attitudes mediate the relationship between digital health literacy and health anxiety among older adults.

H3: Physical activity mediates the relationship between digital health literacy and health anxiety among older adults.

H4: Aging attitudes and physical activity sequentially mediate the relationship between digital health literacy and health anxiety among older adults.

## Method

### Participants

This study adopted a cross-sectional survey design and recruited older adults aged 60 years and above living in mainland China. Using a convenience sampling approach, participants were recruited from community elderly service centers, neighborhood committee activity stations, universities for older adults, and primary healthcare service centers located in Jiangsu, Shandong, and Shanghai. Eligible participants were those aged ≥ 60, residing in mainland China for at least 6 months, capable of basic communication, and willing to participate; individuals with severe cognitive impairment, acute psychiatric symptoms, or acute-stage severe illness were excluded. Before data collection, all participants were informed of the study purpose and provided written consent. A total of 600 questionnaires were distributed, and after removing invalid responses (e.g., uniform answering patterns or substantial missing data), 535 valid questionnaires were retained, yielding an effective response rate of 89.17%. The final sample included 298 men and 237 women, with 268 participants aged 60–69 years, 187 aged 70–79 years, and 80 aged 80 years or above. Educational attainment included primary school or below (*n* = 161), junior high school (*n* = 187), senior high/technical secondary school (*n* = 107), and junior college or above (*n* = 80). Marital status comprised married (*n* = 375), widowed (*n* = 107), and divorced or unmarried (*n* = 53). A total of 321 participants resided in urban areas and 214 in rural areas. Daily internet use varied, with 134 reporting <1 h, 187 reporting 1–3 h, 134 reporting 3–6 h, and 80 reporting >6 h.

## Measures

### Demographic variables

Demographic information was collected, including age, gender, educational attainment, marital status, place of residence (urban/rural), and daily internet use time. These variables were included as control variables in all regression and mediation analyses to reduce the potential confounding effects of demographic factors on the study results.

### Digital health literacy

Digital health literacy was assessed using the Digital Health Literacy Scale developed by Norman and Skinner (10), which has been widely used in international research. The scale was subsequently translated and culturally adapted for the Chinese context by Guo et al. (43), who also validated its applicability in Chinese populations. The scale consists of eight items encompassing three dimensions: the ability to apply online health information and services (e.g., “I know how to find useful health resources on the Internet”), evaluation ability (e.g., “I have the skills to judge the quality of online health resources”), and decision-making ability (e.g., “I feel confident using information from the Internet to make health-related decisions”). All items were rated on a five-point Likert scale ranging from 1 (“strongly inconsistent”) to 5 (“strongly consistent”). In the present study, the scale demonstrated excellent internal consistency (Cronbach’s *α* = 0.956). Confirmatory factor analysis (CFA) indicated a good model fit (χ^2^/df = 2.097, NFI = 0.976, TLI = 0.997, CFI = 0.989, RMSEA = 0.043), supporting the reliability and validity of the scale in this sample.

### Health anxiety

Health anxiety was measured using the short version of the Health Anxiety Inventory (HAI) developed by Salkovskis et al. (44), which has demonstrated good reliability and validity in assessing health anxiety. The scale was translated and adapted into Chinese by Zhang et al. (45), who confirmed its applicability in Chinese populations. The Chinese version uses a four-point Likert scale, with response options ranging from 1 (“never”) to 4 (“always”). In the present study, the scale showed excellent internal consistency (Cronbach’s *α* = 0.973). CFA results indicated satisfactory model fit (χ^2^/df = 2.103, NFI = 0.930, TLI = 0.982, CFI = 0.983, RMSEA = 0.043), suggesting good reliability and validity.

### Aging attitudes

Aging attitudes were assessed using the short form of the Attitudes to Aging Questionnaire (AAQ-SF) developed by Laidlaw et al. (46). This instrument was translated into Chinese by Wang and Li (47) and has been widely applied among older adults in China. The scale comprises 12 items across three dimensions: psychological growth (e.g., “Growing older has been a positive experience”), psychosocial loss (e.g., “Old age is a time of loneliness”), and physical change (e.g., “I have more energy now than I expected for my age”). Responses were rated on a five-point Likert scale ranging from 1 (“completely incorrect”) to 5 (“completely correct”). In this study, the scale exhibited high internal consistency (Cronbach’s *α* = 0.967). CFA results demonstrated good model fit (χ^2^/df = 2.414, NFI = 0.961, TLI = 0.985, CFI = 0.988, RMSEA = 0.038), indicating satisfactory reliability and validity.

### Physical activity

Physical activity was measured using the Physical Activity Rating Scale originally developed by Kimio (48). This scale evaluates physical activity levels across three dimensions: exercise intensity, frequency, and duration. Items are rated on a five-point Likert scale, and an overall physical activity score is calculated using the formula: Physical activity score = exercise frequency × (exercise duration − 1) × exercise intensity, with total scores ranging from 0 to 100. The applicability of this instrument among Chinese older adults have been well established in previous research (49).

### Data analysis

Data analyses were conducted in three steps. First, internal consistency and structural validity of all measurement scales were assessed using Cronbach’s alpha coefficients and confirmatory factor analysis, respectively. Second, Harman’s single-factor test was employed to examine the presence of potential common method bias. Finally, for hypothesis testing, mediation analyses were performed using the PROCESS macro developed by Hayes within SPSS 26.0. Specifically, the bootstrap method with 5,000 resamples was applied to generate the sampling distribution of indirect effects, thereby improving the robustness of the estimates. For each resample, path coefficients of direct and indirect effects were calculated, and statistical significance was determined based on the 95% confidence intervals (CIs); an effect was considered significant if the CI did not include zero. This analytical approach reduces sampling error and enhances the reliability of statistical inferences (50).

## Results

### Common method Bias

To minimize the potential influence of common method bias, several procedural remedies were implemented during data collection. Specifically, participants were informed that the questionnaire was intended solely for academic research purposes and that there were no right or wrong answers. Anonymity was assured to reduce social desirability effects. In addition, items measuring different constructs were randomly ordered and interspersed to prevent response patterns caused by item adjacency. Several reverse-coded items were also included to enhance response discrimination and reduce the likelihood of mechanical responding. Collectively, these procedural measures were expected to mitigate the risk of common method bias. Furthermore, Harman’s single-factor test was conducted as a statistical assessment of common method bias (51). The results indicated that the largest single factor accounted for 27.65% of the total variance, which is below the commonly accepted threshold of 40%. This finding suggests that no single factor dominated the variance structure of the data, indicating that common method bias was not a serious concern in the present study.

### Correlation analysis

As shown in Table 1, after controlling for demographic variables including gender, age, education level, and marital status, the correlation analysis indicated that digital health literacy was significantly positively correlated with aging attitudes (*r* = 0.233, *p* < 0.01) and physical activity (*r* = 0.169, *p* < 0.01). Aging attitudes were also significantly positively correlated with physical activity (*r* = 0.181, *p* < 0.01). Meanwhile, health anxiety was significantly negatively correlated with digital health literacy (*r* = −0.193, *p* < 0.01), aging attitudes (*r* = −0.258, *p* < 0.01), and physical activity (*r* = −0.142, *p* < 0.01).

**Table 1:** Correlation analysis.

Variables	Mean	SD	1	2	3	4
1. DHL	24.00	6.89	1			
2. AA	36.13	10.28	0.233**	1		
3. PA	22.19	24.56	0.169**	0.181**	1	
4. HA	18.11	8.52	−0.193**	−0.258**	−0.142**	1

DHL, digital health literacy; HA, health anxiety; AA, aging attitudes; PA, physical activity; *p < 0.05, **p < 0.01.

### Mediation analysis

As shown in Tables 2, 3, after controlling for demographic variables including gender, age, education level, marital status, place of residence, and daily internet use, the path analyses revealed that digital health literacy remained significantly negatively associated with health anxiety when no mediators were included (*β* = −0.239, *p* < 0.01), supporting Hypothesis 1. After the mediators were entered into the model, digital health literacy showed significant positive associations with aging attitudes (*β* = 0.351, *p* < 0.01) and physical activity (*β* = 0.464, *p* < 0.01). In turn, aging attitudes (*β* = −0.174, *p* < 0.01) and physical activity (*β* = −0.030, *p* < 0.05) were significantly negatively associated with health anxiety. The direct association between digital health literacy and health anxiety remained significant (*β* = −0.161, *p* < 0.01) even after including the mediators.

**Table 2:** Mediation effect test.

Variables	AA			PA			HA			HA		
	*β*	*SE*	*t*	*β*	*SE*	*t*	*β*	*SE*	*t*	*β*	*SE*	*t*
Gender	2.457	2.601	0.944	0.812	6.236	0.13	1.6	2.176	0.735	2.079	2.12	0.981
Age	−0.095	1.56	−0.061	−2.893	3.738	−0.774	0.345	1.305	0.264	0.241	1.271	0.19
EL	0.299	0.574	0.521	−2.479	1.376	−1.802	−0.557	0.48	−1.159	−0.575	0.469	−1.226
MS	−0.782	1.015	−0.77	−0.849	2.434	−0.349	−0.163	0.85	−0.191	−0.333	0.827	−0.402
POR	−0.969	2.525	−0.384	−5.984	6.05	−0.989	−1.781	2.112	−0.843	−2.138	2.058	−1.039
IUT	0.037	0.59	0.062	−0.277	1.414	−0.196	0.233	0.494	0.473	0.232	0.481	0.483
DHDL	0.351**	0.063	5.569	0.464**	0.155	2.985	−0.239**	0.053	−4.54	−0.161**	0.053	−3.017
AA				0.363**	0.104	3.482				−0.174**	0.036	−4.859
PA										−0.030*	0.015	−2.001
*R*^2^	0.063			0.059			0.044			0.098		
Adjust *R*^2^	0.051			0.045			0.031			0.082		
F	*F* (7,527) = 5.066,p = 0.000			*F* (8,526) = 4.140,*p* = 0.000			F (7,527) = 3.455,*p* = 0.001			*F* (9,525) = 6.307,p = 0.000		

EL, educational level; MS, marital status, POR, place of residence; IUT, internet usage time; DHL, digital health literacy; HA, health anxiety; AA, aging attitudes; PA, physical activity; *p < 0.05, **p < 0.01.

**Table 3:** Bootstrap method for mediating effect testing.

Path	Effect	Boot SE	Boot LLCI	Boot ULCI
DHDL→AA→HA	−0.061	0.014	−0.08	−0.025
DHDL→PA→HA	−0.014	0.007	−0.029	−0.002
DHDL→AA→PA→HA	−0.004	0.002	−0.008	−0.001

DHL, digital health literacy; HA, health anxiety; AA, aging attitudes; PA, physical activity.

Finally, bias-corrected bootstrap analyses (5,000 resamples) were conducted to further examine the mediating roles of aging attitudes and physical activity. Results indicated that the indirect effect of aging attitudes was −0.061 [Boot SE = 0.014, 95% CI (−0.080, −0.025)], and the indirect effect of physical activity was −0.014 [Boot SE = 0.007, 95% CI (−0.029, −0.002)]. The sequential mediating effect of aging attitudes followed by physical activity was −0.004 [Boot SE = 0.002, 95% CI (−0.008, −0.001)]. All confidence intervals excluded zero, indicating that all three mediation pathways were statistically significant, thereby supporting Hypotheses 2, 3, and 4.

## Discussion

### Digital health literacy and health anxiety among older adults

The results of this study indicate a significant negative association between digital health literacy and health anxiety among older adults, thereby supporting Hypothesis 1. This finding suggests that digital health literacy plays a protective role in alleviating health anxiety in later life. Older adults with higher levels of digital health literacy are better able to access, screen, and evaluate online health information, which reduces cognitive burden and informational uncertainty when confronting illness and health-related risks. When judgments are based on reliable and comprehensible information, the tendency to catastrophize bodily sensations decreases, leading to lower levels of anxiety (6, 10). This association can be interpreted through the lens of Conservation of Resources theory. Digital health literacy provides older adults with the capacity to identify and integrate health-related information, constituting an important form of cognitive resource. The accumulation of such resources enables individuals to maintain a sense of informational control and psychological balance when facing health threats, thereby buffering anxiety responses. In contrast, older adults with lower digital health literacy often lack effective skills for evaluating online health information and are therefore more vulnerable to misleading, exaggerated, or fragmented health messages, which may amplify perceived health threats and increase anxiety levels (22). Moreover, substantial disparities in educational attainment, urban–rural background, and generational experiences are prevalent among older adults in China, creating relatively high barriers to the effective use of digital tools (13). Improving digital health literacy may help reduce resource inequalities arising from gaps in knowledge and technology, facilitating a shift from passive dependence on healthcare services to more active engagement in health management (52). This role transition reflects a process of psychological resource reconstruction, characterized by enhanced perceived control and agency, which may further contribute to anxiety reduction (53, 54).

Notably, the present findings are consistent with recent international research. Dağ et al. (55) reported a similarly significant negative association between digital health literacy and health anxiety among university students in health-related disciplines, suggesting that digital health literacy may function as a psychological protective factor across different cultural and age groups. By extending this line of inquiry to a Chinese older adult population, the current study enhances the external validity of existing evidence and underscores the broader relevance of digital health literacy across diverse demographic contexts.

Taken together, these findings suggest that, within the framework of the “Healthy China” initiative, digital health education should be integrated into strategies addressing population aging, with particular attention to older adults in rural areas and those with lower levels of education. At the community and family levels, the development of age-friendly digital health platforms and tiered digital training programs may help enhance older adults’ cognitive resources and reduce anxiety risks stemming from information asymmetry and resource constraints.

### The mediating role of aging attitudes

The present study found that aging attitudes mediated the relationship between digital health literacy and health anxiety among older adults, thereby supporting Hypothesis 2. This finding reveals a potential psychological pathway through which digital health literacy influences health anxiety in later life. Older adults with higher levels of digital health literacy are more likely to access trustworthy health information sources and to demonstrate stronger abilities to evaluate and select information when confronted with large volumes of online content (56). As a result, they are better able to acquire accurate, science-based information related to aging and to identify misleading content or negative social narratives (57). These information-processing capabilities help older adults maintain a more rational perspective when facing stereotypes that equate aging with inevitable decline, thereby reducing the internalization of negative beliefs and facilitating the development of a “positive aging” cognitive framework. Previous research has shown that individuals with higher levels of health information literacy are more inclined to view aging as a stage of experience accumulation and continued social contribution rather than as a process defined primarily by physical deterioration (58). Importantly, positive aging attitudes represent not only a cognitive orientation but also a valuable psychological resource. Such attitudes enable individuals to approach health-related threats with greater emotional balance, reducing catastrophic interpretations of bodily changes and subsequent anxiety responses (59). When older adults hold positive views of aging, they are more likely to interpret physical changes as a natural part of the life course rather than as early signs of illness or imminent health risks. This perspective helps reduce excessive worry about future health problems and, in turn, alleviates anxiety. In contrast, older adults with lower digital health literacy may be more susceptible to negative aging narratives or health-related misinformation, which can foster negative aging attitudes, amplify catastrophic appraisals of health risks, and exacerbate health anxiety.

This mediating mechanism appears particularly salient within the Chinese cultural context. Influenced simultaneously by Confucian values emphasizing respect for older adults and by modern discourses portraying population aging as a social burden, older adults often occupy a contradictory position in social evaluations (60, 61). On the one hand, they are expected to serve as carriers of family wisdom and experience; on the other hand, negative narratives equating old age with decline remain widespread (62). Such tensions may lead older adults to internalize negative self-perceptions of aging, thereby increasing vulnerability to adverse psychological outcomes (63). However, higher levels of digital health literacy may enable older adults to access and adopt emerging concepts such as “positive aging” and “successful aging,” allowing them to develop more favorable self-perceptions amid sociocultural pressures (64).

Taken together, these findings suggest that digital health literacy is not only directly associated with health anxiety among older adults but also exerts an indirect effect through improvements in aging attitudes. This highlights the need for public health interventions to move beyond approaches that focus solely on psychological counseling or health education. Instead, greater emphasis should be placed on integrating digital skills training with the promotion of positive aging concepts. For example, community-based educational programs should not only teach older adults how to use digital tools to access reliable health information but also actively disseminate positive aging narratives to gradually reduce negative perceptions of aging. At the same time, it should be acknowledged that the implementation of such interventions may be constrained by factors such as technology acceptance, urban–rural disparities, and resource availability, underscoring the importance of context-sensitive and phased approaches. Overall, by confirming the mediating role of aging attitudes, this study elucidates a key psychological pathway through which digital health literacy alleviates health anxiety and provides meaningful implications for promoting mental health among older adults.

### The mediating role of physical activity

The present study further revealed that physical activity significantly mediated the relationship between digital health literacy and health anxiety among older adults, thereby supporting Hypothesis 3. This finding indicates that digital health literacy is not only directly associated with health anxiety but also indirectly alleviates anxiety by promoting engagement in physical activity. Older adults with higher levels of digital health literacy are more likely to access scientific and personalized exercise-related information, which facilitates the development of more proactive and regular exercise habits in daily life (65). Engagement in physical activity contributes to anxiety reduction through multiple pathways. At the physiological level, regular exercise has been shown to improve cardiovascular and neural functioning (66, 67). At the psychological level, physical activity enhances emotion regulation capacity and self-efficacy, both of which are closely associated with lower levels of anxiety (68). In addition, participation in physical activity often provides opportunities for social interaction, increasing social support and a sense of belonging among older adults (69). These social resources further strengthen psychological resilience and stress-coping capacity (70). Importantly, the mediating role of physical activity extends beyond its physiological benefits and reflects a broader process of resource accumulation. Through sustained participation in physical activity, older adults can experience positive emotions and engage in meaningful social interactions within exercise contexts (71). The continuous accumulation of these physical, psychological, and social resources contributes to enhanced resilience and greater confidence in emotion regulation (72). This finding highlights a resource transformation mechanism in older adults’ health behaviors, whereby cognitive resources at the informational level (e.g., the ability to understand and evaluate health information) are converted into new psychological and social resources through behavioral investment (e.g., regular physical activity) (26, 73), ultimately fostering a positive cycle of mental health.

Taken together, these results suggest that future public health interventions should integrate digital health education with physical activity promotion. On the one hand, older adults should be supported in developing skills to critically evaluate and apply health information, enabling them to select and adopt scientific and feasible exercise practices. On the other hand, community- and policy-level initiatives should expand access to physical activity spaces and strengthen social support networks, particularly for older adults in rural areas, those with lower educational attainment, or those living alone. By combining improvements in digital health literacy with the promotion of physical activity, it may be possible to reduce health inequalities while simultaneously lowering the risk of health anxiety among older adults.

### The chain mediating role of aging attitudes and physical activity

The present study further demonstrated that the relationship between digital health literacy and health anxiety operates not only through the independent mediating effects of aging attitudes and physical activity, but also through a sequential mediating pathway. Specifically, higher digital health literacy was associated with more positive aging attitudes, which in turn were related to higher levels of physical activity and, ultimately, to lower levels of health anxiety. This finding provides a more nuanced framework for understanding how digital health literacy is translated into concrete psychological and behavioral protective factors in later life.

First, the results suggest that positive cognitive perceptions of aging can be transformed into health-related behaviors, and that the cumulative effects of these behaviors are reflected in reduced anxiety. In this sense, positive aging attitudes should not be regarded as static beliefs, but rather as dynamic cognitions that reshape older adults’ perceptions of their physical capabilities and future expectations (15). Such attitudes motivate individuals to engage more proactively in health-maintaining behaviors, including regular physical activity (74). Positive aging attitudes have been shown to enhance self-efficacy (75) and to strengthen individuals’ recognition of the value of maintaining physical functioning (59). These psychological resources, in turn, guide older adults toward more active participation in physical activity (39, 76) and encourage them to view exercise as a worthwhile form of self-care (77).

Second, higher levels of physical activity are consistently associated with lower levels of anxiety. Existing studies indicate that regular low- to moderate-intensity physical activity is linked to greater subjective well-being (78) and improved sleep quality (79). When situated within the Conservation of Resources framework, this process can be conceptualized as a chain of resource accumulation (26). Digital health literacy provides a form of cognitive resource that enables older adults to effectively access and evaluate health-related information (80), thereby fostering positive aging attitudes. These attitudes function as psychological resources that help individuals maintain a sense of self-worth and control during the aging process (15). Subsequently, such psychological resources are converted into behavioral resources in the form of sustained physical activity. Physical activity then contributes to anxiety reduction through physiological regulation and enhanced emotion regulation resources (81), ultimately lowering levels of health anxiety. Taken together, this sequential pathway illustrates how digital health literacy indirectly influences health anxiety through interconnected psychological and behavioral resources, thereby deepening our understanding of older adults’ psychological adjustment processes in digital health contexts.

Notably, this chain mediation effect may be particularly salient among older adults in China. Within the cultural context of filial piety, filial responsibility is not only understood as an obligation of adult children to care for their parents, but is also increasingly internalized by older adults as a form of self-imposed health responsibility. Specifically, maintaining physical functioning and independence is perceived as a way to avoid becoming a burden on one’s children (82). As a result, positive aging attitudes in this context reflect not only favorable perceptions of aging, but also an active commitment to fulfilling family responsibilities. Previous research has shown that when older adults view aging as a life stage in which functional capacity can still be preserved, personal value sustained, and family responsibilities fulfilled through individual effort, positive aging attitudes are more likely to be translated into concrete health behaviors, such as physical activity (38). In this process, physical activity is not merely seen as a means of improving physical fitness or delaying decline, but as an important strategy for maintaining self-care ability and reducing family burden. Consequently, older adults are more inclined to regard physical activity as a long-term self-management behavior. This interpretation is consistent with prior findings showing that positive self-perceptions of aging are more readily expressed through health behaviors such as physical activity (74), thereby reducing excessive worry about future health uncertainty and alleviating health anxiety.

In addition, the present findings suggest that single-dimension health education interventions may have limited long-term effectiveness in promoting older adults’ mental health. Teaching exercise techniques alone, or providing instruction solely on how to evaluate health information, is unlikely to simultaneously change older adults’ health-related cognitions, attitudes, and behaviors. Therefore, integrated intervention strategies are warranted. On the one hand, enhancing digital health literacy can help older adults accurately understand health information and reduce catastrophic interpretations, thereby alleviating health anxiety at the cognitive level. On the other hand, psychological education and social participation programs can support older adults in developing more positive understandings of aging, strengthening self-efficacy and perceived control over health. Finally, improvements in cognition and attitudes should be translated into concrete, feasible, and low-risk physical activity behaviors. Such a comprehensive intervention model, progressing from cognition to attitudes and then to behavior, is more likely to produce sustained buffering effects against health anxiety in older adults.

### Limitations and future directions

Despite providing initial insights into the relationship between digital health literacy and health anxiety among older adults and the underlying mechanisms, this study has several limitations that should be acknowledged.

First, with regard to sampling, a convenience sampling strategy was employed, and participants were primarily recruited from eastern regions of China, including Jiangsu Province, Shandong Province, and Shanghai. As a result, the sample may have limited diversity in terms of regional distribution, urban–rural background, and educational attainment, which could restrict the external validity of the findings. Future studies should consider adopting stratified or random sampling approaches and expanding recruitment to include older adults from different regions, residential contexts, and socioeconomic backgrounds to enhance representativeness and generalizability.

Second, the present study adopted a cross-sectional design. Although the mediation analyses provided support for the proposed theoretical model, such a design limits the ability to draw firm causal inferences. Because all variables were measured at a single time point, the temporal ordering and dynamic relationships among digital health literacy, aging attitudes, physical activity, and health anxiety could not be clearly established. Future research should employ longitudinal designs with multiple measurement waves to capture changes over time and to more rigorously test causal relationships among these variables.

Third, in terms of measurement, this study relied primarily on self-report questionnaires to assess key constructs, including physical activity and health anxiety. While self-report measures are widely used and practical, they are susceptible to social desirability bias and recall bias, which may compromise measurement accuracy and internal validity. Future studies could incorporate more objective and multimethod assessments, such as wearable devices to record physical activity or physiological indicators collected in clinical settings, to strengthen the robustness of the findings.

Finally, regarding variable selection, this study focused on aging attitudes and physical activity as mediators but did not include other potentially important factors. Variables such as social support, family intergenerational interactions, and broader social contexts may also play meaningful roles in the association between digital health literacy and health anxiety. Future research may benefit from developing more comprehensive conceptual models that integrate these additional factors to further elucidate the complex mechanisms linking digital health literacy to health anxiety in older adults.

## Conclusion

This study found that higher levels of digital health literacy were negatively associated with health anxiety among older adults. Importantly, this association was not limited to a direct relationship but also operated indirectly through aging attitudes and physical activity. Specifically, older adults with higher digital health literacy tended to report more positive aging attitudes and higher levels of physical activity, both of which were significantly associated with lower health anxiety. These findings suggest that efforts to alleviate health anxiety in older adults should adopt an integrated approach that simultaneously emphasizes digital health education, physical activity promotion, and the cultivation of positive aging perspectives.

## Data Availability

The raw data supporting the conclusions of this article will be made available by the authors, without undue reservation.
